# Nurse’s Sore Mouth

**Published:** 1849-09

**Authors:** J. Yale Ware

**Affiliations:** Mass.


*Nurses' Sore Mouth.* By J. Yale Ware, Mass.—This is a very troublesome complaint of mothers, and one that is rapidly increasing in this section. An infallible remedy when it does not purge the bowels, is Griffitt's Myrrh Mixture, in tablespoonful doses three times a day, internally, and a solution of nitrate of silver gr. viii to ℥iv of pure water for a gargle, and, if the soreness extends towards the stomach, swallow a teaspoonful three times a day. Of course, if need be, the stomach should be cleansed by a light emetic or gentle laxative, antecedent to the above treatment. I believe, if perseveringly used in season, very few mothers need wean their children from this cause; indeed, I have never known it fail to relieve the mouth.—*Am. Jour. of Med. Sciences.*

